# Effect of ivabradine on cardiovascular outcomes in patients with stable angina: meta-analysis of randomized clinical trials

**DOI:** 10.1186/s12872-017-0540-3

**Published:** 2017-04-28

**Authors:** Hayelom Gebrekirstos Mengesha, Berhe Weldearegawi, Pammala Petrucka, Tadese Bekele, Mala George Otieno, Abraha Hailu

**Affiliations:** 1 College of Health Science, Adigrat University, Adigrat, Ethiopia; 2 College of Health Science, School of Public Health, Mekelle University, Mekelle, Ethiopia; 3 College of Nursing; Adjunct Nelson Mandela African Institute of Science and Technology, University of Saskatchewan, Saskatoon, Canada; 4 College of Health Science, Department of Pharmacy, Haramaya University, Harar, Ethiopia; 5 College of Health Science, Department of Medical Biochemistry, Mekelle University, Mekelle, Ethiopia; 6 College of Health Science, Department of Internal Medicine, Mekelle University, Mekelle, Ethiopia

**Keywords:** Ivabradine, Randomized trials, Meta-analysis, Funny current, Coronary artery disease

## Abstract

**Background:**

Although there are established drugs for treatment of cardiovascular diseases, due to adverse effects these drugs may not be clinically applicable to all patients. Recent trends have seen the emergence of drugs which act on funny current channels to induce selective heart rate reduction. Ivabradine is one such drug developed for coronary artery disease and heart failure. There is inconsistent evidence about the effect of this selective inhibitor in reduction of cardiovascular related mortality and morbidity. Such an inconsistency warrants the need for a meta-analysis to consider the effectiveness and efficacy of Ivabradine in the treatment of coronary artery disease and heart failure.

**Methods:**

Randomized controlled trials with a minimum follow-up period of one year were searched in Pub Med/Medline, Embase, Cochrane Central Register of Controlled Trials published between 1980 and 2016.Each eligible study was assessed for risk of bias by using the Cochrane Risk of Bias Assessment tool. The outcomes assessed in this study included: all cause mortality, cardiovascular-related mortality, hospitalization for new or worsening heart failure, and adverse events. Subgroup analysis and publication bias were assessed. We used Mantel-Haenszel method for random-effects. Analysis was done using RevMan5.1™.This study was registered in PROSPERO as [PROSPERO 2016:CRD42016035597].

**Result:**

Three trials with a total of 36,577 participants met the meta-analysis criteria. Pooled analysis showed that ivabradine is not effective in reducing cardiovascular deaths (OR: 1.02; CI:0.91–1.15,*P* = 0.74), all-cause mortality (OR:1.00; CI:0.91–1.10,*P* = 0.98), coronary revascularization (OR: 0.93, CI: 0.77–1.11, *P* = 0.41) and hospital admission for worsening of heart failure (OR: 0.94, CI: 0.71–1.25, *P* = 0.69). However, the drug was found to significantly increase adverse events: phosphenes (OR:7.77, CI: 4.4–14.6,*P* < 0.00001), blurred vision (OR:3.07,CI:2.18–4.32,*P* < 0.00001), symptomatic bradycardia (OR: 6.23, CI: 4.2–9.26, *P* < 0.00001), and atrial fibrillation (OR: 1.35, CI: 1.19–1.53, *P* < 0.0001). Subgroup analysis by duration of follow up on cardiovascular outcomes found that there is no difference in effect of ivabradine depending on the duration of follow up. There was no publication bias in reporting of included studies.

**Conclusion:**

This meta-analysis suggests that ivabradine is not effective in reducing cardiovascular-related morbidity and mortality unless used for specific conditions. On the contrary, the use of this drug was strongly associated with the onset of untoward and new adverse events. This finding strongly supports previous findings and further informs the rational and evidence-informed clinical use of ivabradine.

**Electronic supplementary material:**

The online version of this article (doi:10.1186/s12872-017-0540-3) contains supplementary material, which is available to authorized users.

## Background

Globally, cardiovascular diseases (CVD), generally, and coronary artery disease (CAD) is the leading cause of death in developed contexts and is emerging as a leading cause in developing countries [1]. For populations over 45 years of age in 2020, it is estimated that CAD will be responsible for a total of 11.1 million annual deaths globally [2].

To reduce the burden of CVD morbidities and mortality, a range of preventive and therapeutic interventions exist. Previous studies suggest that the major established risk factors for CVD include smoking, hypertension, obesity, diet, and harmful use of alcohol, amongs to thers [3, 4]. In addition to these major established risk factors, a recent follow-up epidemiologic study showed that resting heart rate is a predictor of CVD morbidity and mortality [5]. Increased heart rate independent of other cardiovascular diseases or risk factors has been associated with atherosclerosis, heart failure, coronary artery disease, hypertension, and stroke [6–9].

A number of preventive therapies have been developed to prevent the onset and complications of CAD [10–12]. Different classes of medications, such as beta-blockers, calcium channel blockers, and nitrates, reduce the heart rate, thereby reducing mortality risk attributable to higher heart rates [10–12]. Although these classes of drugs have clinical uses in many CVD, they lack of selectivity and specificity for the reduction of heart rate and are frequently associated with adverse effects [10–13].

The limitations of these classes of drugs led to investigation of the novel site useful for “selective or pure” reduction of heart rate called funny current channel(I*f*.)With the recognition that pacemaker current is the modulating attribute, one of the first medications designed, tested, and implemented to inhibit the I*f* channel of the sino-atrial node [13] was Ivabradine. Randomized controlled trials on Ivabradine, such as the BEAUTIFUL(mor**B**idity-mortality **E**v**A**l**U**a**T**ion of the **If** inhibitor ivabradine in patients with coronary disease and left-ventric**UL**ar dysfunction), SHIFT(**S**ystolic **H**eart failure treatment with the **If** inhibitor ivabradine **T**rial), and SIGNIFY(Study Assessing the Morbidity–Mortality Benefits of the I*f* Inhibitor Ivabradine in Patients with Coronary Artery Disease), considered patients with heart failure and stable CAD [9, 14, 15]. Some studies found protective effects of ivabradine mainly for heart rate ≥ 70 beats per minute (bpm) [11, 15], whilst a recent study found no additional effects of ivabradine for CAD patients [14].

In light of the evidence, ivabradine was approved in Europe for use in patients exhibiting stable CAD accompanied by normal sinus rhythm with contraindications for β-blockers [16] and, based on the 2010 findings of the BEAUTIFUL study [9], for patients with uncontrolled angina symptoms and heart rates in excess of 60 bpm despite β-blocker therapy. In 2012, the drug was approved, in America, for treatment of chronic heart failure (Class II-IV New York Heart Association) following the SHIFT study [15] and, in 2015, heart failure with a heart rate of ≥70 bpm [17]. Previous randomized trials on ivabradine showed that atrial fibrillation [14, 15, 18], excessive bradycardia [14, 15, 18, 19] and phosphenes [14, 15, 18, 20] were the most frequently reported side effects in the trials.

A recent pooled analysis on the effect of ivabradaine in patients with stable angina with or without left ventricular dysfunction showed that unselective use of ivabradine is not supported by the evidence and has been associated with new-onset atrial fibrillation, bradycardia, and drug-related nuisance adverse events [18]. Based on these findings, it is imperative to summarize and synthesize the extant evidence on this medication in relation to use or non-use for stable angina. Therefore, this meta-analysis was undertaken to synthesize and analyze relevant randomized control trials conducted between 1980 and 2016 for the overall effect of ivabradine on stable CAD in relation to cardiovascular-related morbidity and mortality.

## Methods

This study was conducted according to the Recommendations for the Conduct, Reporting, Editing, and Publication of Scholarly Work in Medical Journals, specifically the Preferred Reporting Items for Systematic Reviews and Meta-analysis PRISMA Checklist (Additional file 1) [21].

### Inclusion/exclusion criteria

The study included all accessible randomized double blind, placebo controlled, and non-inferiority studies conducted to determine or compare the effect of ivabradine on stable CAD with heart failure, and without clinical heart failure compared with placebo or standard care. Further, included studies required measured cardiovascular end points, minimum follow up of one year, and occurred within the timeframe of the period of 1980 through January 2016. In order to address risk of bias in accordance with Cochrane Collaboration recommendations, studies with participants that had myocardial infarction or unstable angina before starting treatment and which do not have a clear measure of the outcome of interest were excluded.

### Types of outcome and interventions

The outcomes analyzed were all-cause mortality, cardiovascular related deaths, cardiovascular related hospitalizations, hospitalization for worsening or new onset heart-failure, and coronary revascularization in patients with stable CAD and clinical heart failure. Adverse event outcomes included: trial fibrillation, symptomatic bradycardia, phosphene, cardiac disorders, as well as any other documented serious adverse events or infections.

### Search strategy

We searchedOvid Medline, Pubmed, Embase, Scopus, Clinical Trials.gov and the Cochrane Central Register of Controlled Trials for randomized placebo controlled trials of ivabradine effect using the terms ‘Ivabradine’, ‘procoralan’, “stable coronary artery disease”, ‘stable angina’, ‘ischemic heart disease’, “randomized controlled trial”, and “placebo” as text words, and corresponding MeSH terms. We searched for studies in the reference lists of a meta-analysis study, controlled trials, and review articles from the established review period. Efforts were made to identify, include, and acquire grey literature (i.e., unpublished studies) via personal contacts and/or emails lead by BW and TB.

### Data extraction, measure of effect and analysis

Data was extracted by two independent reviewers using a data extraction template. Where disagreement exists reviewers discussed about the issues to reach consensus. A Mantel-Haenszel random-effect model was used to consider the heterogeneity of studies and calculate combined effect size to provide a more conservative estimation of odds ratio (OR) and 95% confidence interval (CI). When I^2^ < 50 fixed effect model was used. Individual patients were the units of analysis. Missing data was considered by intent to treat analysis using imputation assuming missing data happened at random and would have similar outcomes to the available data.

To assess statistical heterogeneity between summary data at trial level we used the tau statistic (*P* < 0.10) and the I^2^statistic (I^2^ > 50%) was considered low if I^2^ < 30%, moderate if I^2^ is between30 75%, and high if I^2^ > 75% [21]. Publication and other reporting biases were assessed using funnel plots and Egger’s test where necessary. The effect of ivabradine on cardiovascular outcomes by stratifying based on duration of follow up of patients was investigated using random and fixed effects meta-regression analyses.

Analysis was done using Rev. Man 5.1™ and CMA 3.0™. All tests were two tailed and considered significant if *P* < 0.05.

### Assessment of risk of bias in included studies

The risk of bias in this study was assessed using the risk of bias assessment tool for randomized control trials [22]. The Cochrane risk of bias domains was used to identify the risk of bias in individual studies [22]. The domains were: random sequence generation (selection bias), allocation concealment (selection bias), blinding of participants and personnel (performance bias), blinding of outcome assessors (detection bias), incomplete outcome data (attrition bias), selective outcome reporting (reporting bias), academic bias and source of funding bias. Each included study was assessed for each domains, with ratings of low, uncertain, or high risk in accordance with the criteria as published in PROSPERO [23].

### Ethical clearance

Ethical clearance was not necessary for this study.

## Results

### Study selection and characteristics of the included studies

**Fig. 1:**
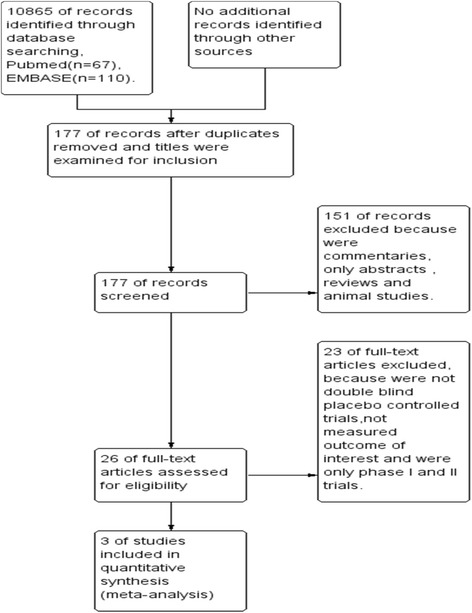
Study flow diagram for the study inclusion and exclusion process

In this analysis a total of 11,042 records were assessed for eligibility and of these 10,865 records were excluded because they were duplicates and after titles were examined. Of 177 records 151 were excluded because they were only abstracts, animal studies, commentaries, and/or reviews. After further screening, three (3) trials met the requirements for inclusion in the current meta-analysis. The major reasons for exclusion were: not double blind, non-human sample, and non-placebo controlled randomized studies. Figure 1 provides a graphical representation of the selection and de-selection process at this preliminary stage.

Three eligible randomized clinical trials were screened into this analysis, which included a total of 36,577 participants (18,297 in the ivabradine group and 18,280 patients in the placebo group). Sample representativeness included patients with stable angina with left ventricular dysfunction in the BEAUTIFUL trial [9], stable CAD without clinical heart failure in the SIGNIFY trial [14], and chronic heart failure with systolic ventricular dysfunction (plus a majority with ischemic heart disease-originated heart failure) in the SHIFT trial [15]. The median follow up period for these studies was less than two years (i.e., BEAUTIFUL(19 months), SHIFT(22 months), and SIGNIFY(27.8 months)). The left ventricular ejection fraction (LVEF) was less than <40%, <35%, and ≥40%, in the BEAUTIFUL, SHIFT, and SIGNIFY studies, respectively. In these included trials, the dose range of ivabradine ranged between 5 mg and 7.5 mg twice daily. The mean pre-treatment resting heart rate across all three studies was ≥70 bpm. The primary composite end point in the three trials was related to cardiovascular death, admission to hospital for new onset or worsening of heart failure, and admission to hospital for fatal and non-fatal myocardial infarction (refer to Table 1).

**Table 1:** Characteristics of included trials

Trial	Method	Participants	Intervention	Outcome	Duration(months)
BEAUTIFUL 2008	Randomized controlled Trial	10,917(5479 assigned to ivabradine and 5438 assigned to placebo) eligible patients who had coronary artery disease and a LVEF of less than 40%	Ivabradine 5–7.5 mg bid	Cardiovascular death or admission to hospital for myocardial infarction or new-onset or worsening heart failure	19
SHIFT 2010	Randomized controlled Trial	6558 patients with symptomatic heart failure and LVEF of 35% or lower, heart rate of 70 bpm or higher (3268 assigned to ivabradine;3290 assigned to placebo group)	Ivabradine 7.5 mg bid	Cardiovascular death or hospital admission for worsening heart failure	22
SIGNIFY 2014	Randomized controlled trial	19,102 patients(9550 assigned to ivabradine and 9552 assigned to placebo) who had both stable coronary artery disease without clinical heart failure, a heart rate of 70 bpm or more and LVEF of ≥40%	Ivabradine 7.5 mg bid	Death from cardiovascular causes or nonfatal myocardial infarction	27.8

*Bpm* beats per minute, *LVEF* Left ventricular ejection fraction, *bid* twice a day

### Risk of bias assessment of the included studies

Based on the Cochrane Collaboration for risk of bias assessment criteria, the three included studies were low risk in terms of the six major domains. The interpretation of low risk for each domain is explained in detail in our proposal published in PROSPERO [23]. However, assessment for other possible sources of other bias is uncertain due to limited articulation in the respective studies (refer to Table 2).

**Table 2:** Risk of bias table assessment result for the included studies

Study	Criteria for risk of bias assessment						
	Random sequence generation	Allocation concealment	Blinding of participants & personnel	Blinding of outcome assessment	Incomplete outcome data	Selective reporting	Other bias
BEAUTIFUL 2008	LR	LR	LR	LR	LR	LR	UR
SHIFT 2010	LR	LR	LR	LR	LR	LR	UR
SIGNIFY 2014	LR	LR	LR	LR	LR	LR	UR

*LR* Low Risk, *UR* Uncertain risk

Regarding the baseline characteristics of patients the mean (±sd) age of patients was 63.5 ± 9 with 63.7 ± 9 in the ivabradine group and 63.4 ± 9.1 in the placebo group. The majority of participants (i.e., 13,905(76.2%)) were men. The mean LVEF was 32.4(5.5), 29(5.19), and 56.4(8.5) for the BEAUTIFUL, SHIFT, and SIGNIFY trials, respectively. With regard to co-morbidities, 7093(38.8%) and 14,319(78.4%) had diabetes mellitus and hypertension in the ivabradine group, respectively. At randomization, 5193(86%) of participants in the ivabradine group and 5201(87%) in the placebo group were taking β-blockers (refer to Table 3).

**Table 3:** Baseline characteristics of participants included in this metaanalysis

Study	BEAUTIFUL 2008	SHIFT 2010	SIGNIFY 2014	Study arm	
				Ivabradine	Placebo
Age(mean ± SD)	65.2(8.5)	60.4(11.3)	65(7.2)	63.7(9)	63.4(9.1)
use of B-blockers					
Yes	9487(87)	5820(89)	7939(81)	5193(86)	5201(87)
Male	9047(83)	4970(76)	13,839(72)	13,951(76.4)	13,905(76.2)
Female	1870(17)	1535(24)	5263(28)	4319(23.6)	4349(23.8)
DM status	4036(37)	1979(30)	8230(43)	7093(38.8)	7152(39.20)
Hypertension	7720(71)	4314(66)	16,466(86)	14,319(78.4)	14,181(77.7)
Heart rate(mean ± SD)	71.5(9.8)	79.8(9.6)	77.1(7)	76(8.7)	76.3(8.93)
Previous stroke	1997(18%)	526(8)	1265(6.6)	627.3(10.9)	633.3(11.2)
LVEF,mean(SD),in %	32.4(5.5)	29(5.19)	56.4(8.5)	39.3(6.4)	39.3(6.4)
BMI,kg/m^2^,mean(SD)	28.5(4.4)	28(5)	28.7(4.6)	28.4(4.7)	28.4(4.7)

*BMI* Body mass index, *SD* Standard Deviation, *LVEF* Left Ventricular Ejection Fraction, *DM* Diabetes Mellitus, all numbers in bracket except those mentioned denotes percentage (%)

### Effect and safety of ivabradine on cardiovascular outcomes

The effect of ivabradine on cardiovascular outcomes was analyzed using the random-effects model of Der Simonian and Laird considering potential heterogeneity. We summarized the effect of ivabradine compared to placebo on cardiovascular outcomes as follows:

### Efficacy of ivabradine on cardiovascular outcomes

**Fig. 2:**
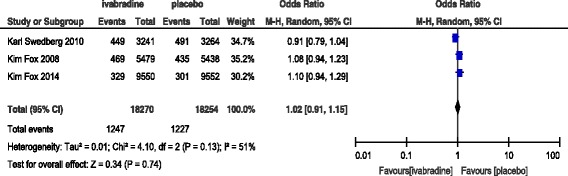
Effect of ivabradine on cardiovascular deaths compared to placebo

**Cardiovascular deaths**: We included three trials with 36,524 patients, which considered cardiovascular deaths as an outcome variable. On meta-analysis, ivabradine did not have a significant effect in reducing cardiovascular caused deaths/events compared to placebo (OR: 1.02; CI: 0.91–1.15, *P* = 0.74). The details of the analysis and forest plot are presented in Fig. 2.

**All-cause mortality**: we assessed all-cause mortality as a one endpoint and found that the effect of ivabradine in reducing all-cause mortality was not different from the placebo (OR: 1.00; CI: 0.91–1.10, *P* = 0.98).

**Admission to hospital for new onset or worsening of heart failure**: all the three trials had assessed this endpoint and were therefore included in the analysis. Ivabradine did not significantly decrease admission to hospital for new onset or worsening of heart failure compared to the placebo (OR: 0.94, CI: 0.71–1.25, *P* = 0.69).

**Coronary revascularization**: Two trials with 30,019 patients assessed the effect of ivabradine on coronary revascularization [14, 15]. Pooled analysis of these trials showed that ivabradine was not effective in reducing the occurrence/events of coronary revascularization compared to placebo (OR: 0.93, CI: 0.77–1.11, *p* = 0.41).

Overall, ivabradine was not found to decrease the number of events related to cardiovascular morbidity and mortality in comparison with placebo group patients with stable angina and heart failure (Table 4).

**Table 4:** Meta-analysis in efficacy and safety of ivabradine in patients with stable coronary angina and heart failure

	Ivabradine		Placebo		Odds ratio	*P*-value	I^2^ in %
Outcome	Event/Total, n/N	%	Event/total	%	M-H, random & Fixed^b^, 95% CI		
Cardiovascular death	1247/18270	6.8	1227/18254	6.7	1.02(0.91–1.15)	0.74	51
All-cause mortality	1560/18270	8.5	1557/18254	8.5	1(0.91–1.10)	0.98	78
Hospital admission^a^	1156/18270	6.3	1280/18254	7	0.94(0.71–1.25)	0.69	89
Coronary revascularization	717/15029	4.7	750/14990	5	0.93(0.77–1.11)	0.41	57
Phosphenes	601/12771	4.7	69/12804	5.3	7.77(4.4–14.6)	<0.00001	78
Symptomatic bradycardia	907/12771	7.1	142/12804	1.1	6.23(4.2–9.26)	<0.00001 1	71
Atrial fibrillation	814/12771	6.3	613/12804	4.7	1.35(1.21–1.51)	<0.00001	22
Blurred vision	134/12771	1	44/12804	0.3	3.07(2.18–4.32)	<0.00001	0
Infection& infestation	519/8709	5.9	555/8690	6.3	0.93(0.82–1.05)	0.26	0
Serious adverse event	2683/8709	31	2792/8690	32	0.97(0.85–1.11)	0.36	48
Cardiac disorder	2203/8709	2.5	2454/8690	2.8	0.85(0.65–1.11)	0.24	89

*CI* Confidence Interval, ^a^ shows hospital admission for worsening or new onset of heart failure,^b^indicates the use of random effect model when I^2^ ≥ 50 and fixed effect if I^2^ < 50

### Safety of ivabradine

All the trials included had assessed the adverse events of ivabradine experienced by study participants. These major adverse events are summarized as follows:

Phosphenes: two trials [14, 15] assessed the adverse effect of ivabradine on vision. Ivabradine significantly increased the incidence of phosphene (OR: 7.77, CI: 4.4–14.6, *P* = <0.00001) and blurred vision (OR: 3.07, CI: 2.18–4.32,*P* = <0.00001) compared to the placebo.

Cardiac: Ivabradine significantly increased the incidence of symptomatic bradycardia (OR: 6.23, CI: 4.2–9.26, *P* = <0.00001) and atrial fibrillation (OR: 1.35, CI: 1.19–1.53) compared to placebo.

In addition.two trials [9, 15] generally assessed the adverse effects of ivabradine on different systems in which incidence of adverse effect was not significantly different on cardiac disorders all serious adverse events, infection and infestation compared to placebo (Table 4).

### Subgroup analysis

A subgroup analysis was undertaken by stratifying the trials based on duration of follow up, reflecting a period of less than two years [9, 15] and one of ≥2 years [14]. Based on this subgroup analysis we found that effect of ivabradine on cardiovascular deaths (OR: 0.99, CI: 0.84–1.17, *P* = 0.9) and all-cause mortality (OR: 0.97, CI: 0.84–1.12, *P* = 0.69) did not significantly vary depending on the duration of follow up by using random effect, Mantel-Haenszel 95% CI analysis.

### Publication bias on the outcome cardiovascular deaths

**Fig. 3:**
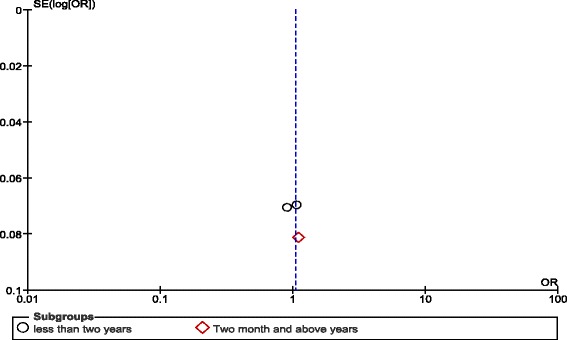
Funnel plot of studies on cardiovascular deaths

**Fig. 4:**
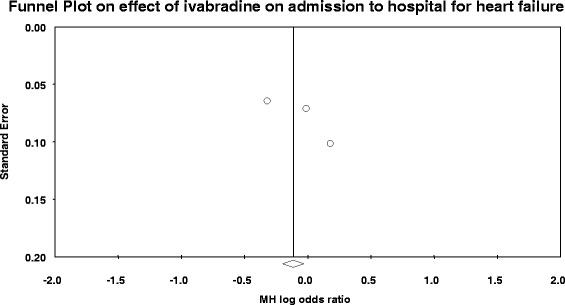
Funnel plot of studies on hospital admission for heart failure

The assessment of publication bias on the three outcomes (cardiovascular, hospital admission for heart failure and all-cause mortality**)** showed that there was no publication bias which could affect the pooled analysis of this study. As can be seen in the funnel plot figures, all the three trials included to this study had no bias in publication. Figures showed that the studies are approximately symmetrically distributed across the line (see Figs. 3 and 4).

Although few number of studies were included to this analysis, Egger’s test to check the publication bias captured by the funnel plot on effect of ivabradine in reducing admission to hospital for worsening or new onset of heart failure found that the intercept (B0) was 0.096, 95% confidence interval (−86.23, 110.13), with *t* = 1.58, df = 1. The one-tailed *p*-value is 0.17, and the two-tailed *p*-value is 0.35. This suggests the absence of significant publication bias.

## Discussion

This study considered the extant evidentiary base on the effects of ivabradine in reducing cardiovascular outcomes in patients with stable angina and heart failure. The research hypothesis was that ivabradine may have a significant effect in reducing cardiovascular outcomes in patients with stable angina and heart failure and that such effect may vary among subgroups and/or across period of follow up. Ivabradine pharmacological effectiveness for these conditions relates to its selective reduction of heart rate by acting on the pacemaker sino-atrial node’s so-called funny channel [13] thereby redressing the established risk factor of increased heart rate [5–9].

A number of large multicenter trials have been conducted; specifically, BEAUTIFUL, SHIFT, and SIGNIFY studies [9, 14, 15]. Although these trials targeted distinct patient populations, they exhibited similarities in terms of the majority of sampled participants exhibiting stable angina and heart failure caused by ischemic heart disease and in terms of the outcomes measured). Specifically, participants in BEAUTIFUL, SHIFT, and SIGNIFY had stable angina with left ventriculardysfunction, heart failure patients (majority with ischemic origin) with ejection fraction <35%, and stable angina without clinical heart failure, respectively. As a result, there was similarity in target population across these studies.

Independently, each trial revealed that ivabradine was effective for reducing events in subgroup patients of heart beat ≥70 bpm in the BEAUTIFUL study and was effective in reducing primary outcome and secondary outcomes in the SHIFT study but in the recent trial with relatively longer duration follow up but without clinical heart failure (SIGNIFY) it was found that the addition of ivabradine to standard background therapy did not improve outcomes [14]. A recent meta-analysis found that unselective use of ivabradine in patients with CAD is not supported by evidence and may be associated with new onset of adverse effects [18]. In the current study, ivabradine was found not to significantly reduce all efficacy outcomes according to the trials assessed in this study. However, in the SHIFT study, ivabradine was found effective in reducing primary composite end point and other secondary outcomes such as hospital admission for worsening of heart failure, but this finding was not true in the pooled analysis for ivabradine compared to placebo participants. Based on this analysis, there is a need to be cautious in interpretation of the effectiveness of ivabradine for patient subgroups with left ventricular dysfunction and high resting heart rate ≥ 70 bpm.

Further, this meta-analysis showed that ivabradine is significantly associated with the new onset of adverse events such as blurred vision, phosphene, atrial fibrillation, and symptomatic bradycardia. Although adverse events varied across the trials, the analysis showed most of the adverse events were significantly related to ivabradine compared to placebo. This finding indicates that the unspecific use (i.e., off label use) of this drug is not effective and may catalyze untoward, adverse events.

There were several limitations in this meta-analysis. Beyond inconsistent diagnostic groups as previously described, some subgroups were too small (i.e., heart rate < 70 bpm versus >70 bpm) and precluded meaningful interpretation of difference. For future we suggest that a pooled analysis is required to estimate the effect size of ivabradine among patients with different heart beats. It was impossible to conduct meta-regression and assess the effect of important variables like heart rate and LVEF which might affect the effect size estimate, because of few numbers of studies included to this meta-analysis.

Subgroup analysis based on duration of follow up, effect of ivabradine did not significantly vary by duration of follow up compared to placebo. This analysis also fills the gap in the hypothesis is that cardiovascular events may vary depending on the duration of follow up.

## Conclusions

On meta-analysis, we found that ivabradine was not effective in reducing cardiovascular morbidity and mortality outcomes but was associated with new onset of adverse events.

In summary, ivabradine is approved by European Medicines Agency [16] and the United States Food and Drug Administrations [17] for clinical use in stable CAD and heart failure of specific conditions. This meta-analysis affirmed that prescribers should be cautious in clinical use of this drug for unspecified conditions or for ‘off label’ purposes. This meta-analysis suggested that ivabradine is not effective in reducing cardiovascular related morbidity and mortality in other than specified conditions and is associated with new onset of adverse events in non-specified use situations. Therefore, this finding strongly supports previous analysis related to the use of ivabradine and emphasizes the imperative for health professionals to be aware of these evidences for rational use of ivabradine in clinical practice.

## Additional file

Additional file 1: PRISMA 2009 Checklist. (DOC 62 kb)

## Abbreviations

BEAUTIFUL: morBidity-mortality EvAlUaTion of the If inhibitor ivabradine in patients with coronary disease and left-ventricULar dysfunction
BPM: Beats per Minute
CAD: Coronary Artery Disease
CMA: Comprehensve MetaAnalysis
CI: Confidence Interval
CVD: Cardiovascular Diseases
PRISMA: Preferred Reporting Items for Systematic Reviews and Meta-analysis PRISMA Checklist
OR: Odds Ratio
SHIFT: **S**ystolic **H**eart failure treatment with the **If** inhibitor ivabradine **T**rial,
SIGNIFY: Study Assessing the Morbidity–Mortality Benefits of the I*f* Inhibitor Ivabradine in Patients with Coronary Artery Disease
